# Unifying view of mechanical and functional hotspots across class A GPCRs

**DOI:** 10.1371/journal.pcbi.1005381

**Published:** 2017-02-03

**Authors:** Luca Ponzoni, Giulia Rossetti, Luca Maggi, Alejandro Giorgetti, Paolo Carloni, Cristian Micheletti

**Affiliations:** 1 SISSA, Trieste, Italy; 2 IAS-5/INM-9: Computational Biomedicine – Institute for Advanced Simulation (IAS) / Institute of Neuroscience and Medicine (INM), Forschungszentrum Jülich, Jülich, Germany; 3 JSC: Division Computational Science – Simulation Laboratory Biology – Jülich Supercomputing Centre (JSC), Forschungszentrum Jülich, Jülich, Germany; 4 JARA-HPC, Jülich, Germany; 5 Department of Oncology, Hematology and Stem Cell Transplantation, University Hospital Aachen, RWTH Aachen University, Aachen, Germany; 6 Department of Biotechnology, University of Verona, Verona, Italy; Weizmann Institute of Science, ISRAEL

## Abstract

G protein-coupled receptors (GPCRs) are the largest superfamily of signaling proteins. Their activation process is accompanied by conformational changes that have not yet been fully uncovered. Here, we carry out a novel comparative analysis of internal structural fluctuations across a variety of receptors from class A GPCRs, which currently has the richest structural coverage. We infer the local mechanical couplings underpinning the receptors’ functional dynamics and finally identify those amino acids whose virtual deletion causes a significant softening of the mechanical network. The relevance of these amino acids is demonstrated by their overlap with those known to be crucial for GPCR function, based on static structural criteria. The differences with the latter set allow us to identify those sites whose functional role is more clearly detected by considering dynamical and mechanical properties. Of these sites with a genuine mechanical/dynamical character, the top ranking is amino acid 7x52, a previously unexplored, and experimentally verifiable key site for GPCR conformational response to ligand binding.

## Author summary

The biological functionality of several receptors and enzymes depends on their capability to sustain large-scale structural fluctuations and adopt different conformational states in response to ligand binding. This is the case for G protein-coupled receptors (GPCRs), the largest superfamily of signaling proteins in mammals and a primary pharmaceutical target. To better understand the functional dynamics of GPCRs, we have analysed the inter-residue distance variations across the available structures for several receptors of the rhodopsin-like family (class A). We first reconstructed the network of mechanical, rigid-like couplings between nearby amino acids and then identified those acting as dynamical/mechanical hubs. These were the sites whose virtual removal led to a significant softening of the overall mechanical network. After validating the biological relevance of these sites by comparison against known key functional sites, we singled out those regions which emerge as prominent mechanical hubs and yet have an otherwise still unknown functional role. The most relevant of such novel putative functional sites, which could be probed by mutagenesis experiments, is at interface of two transmembrane helices and we expect it to be crucial for assisting GPCRs conformational response to agonist binding.

## Introduction

Mammalian G protein-coupled receptors (GPCRs) are the largest family of signaling proteins, with approximately ∼850 unique members up to now identified in the human genome [1, 2]. Given the size of this family, their ubiquitous expression, and their involvement in virtually every (patho)physiological process in mammals, it is not surprising that human GPCRs are targeted by more than half of current drugs [3].

GPCRs share a distinctive structural signature, namely seven *α*-helical transmembrane (TM) domains [4]. Such common structural organization strongly contrasts with the structural diversity of the agonists: these range from subatomic particles (a photon), to ions (H^+^ and Ca^++^), to small organic molecules, to peptides and proteins [4]. The presence of an agonist (or a photon in the case of rhodopsin) triggers specific downstream G protein-dependent signaling pathways.

The mechanisms that precisely control GPCR agonist binding and the following receptor activation have until very recently been hindered by a lack of crystallized active receptor states and receptor-ligand complexes. However, significant advances in crystallization has recently permitted the structural determination of several class A receptors in active state. Moreover, several mutagenesis and assay procedures were performed in an attempt to identify functionally important residues [5], along with specific micro-switches, i.e. small groups of residues that undergo conformational change during receptor activation [6, 7].

Despite a consolidated list of residues important for binding and/or function emerged, the findings are limited by their individualized nature [8].

Indeed, GPCRs are not rigidly switching between the alternative agonist-bound and inactive forms. They rather adopt a series of intermediate conformations influenced not only by association with ligands, but also by other receptors, signaling and regulatory proteins, by post-translational modifications, and by environmental cues [2]. The capability of GPCRs to engage with such diverse signaling machinery strongly depends on their conformational flexibility. All these diverse signaling events are indeed accompanied by dynamic conformational changes. Each state is likely represented by an ensemble of conformations [9].

A characterization of the conformational and structural dynamics of these proteins is therefore critical for understanding the molecular mechanisms underlying their function. A suitable comparative analysis of the available structures for these receptors ought to give insight into their structure–function relationship by clarifying the functional-oriented character of their internal dynamics [10].

While the inspection of GPCRs’ and G proteins’ structures has been essential to map out the accessible distinct signaling states, our knowledge is still limited regarding the internal dynamics of such states and the pathways that link them [11].

To our knowledge this problem has not yet been addressed systematically. The reason for its challenging character lies, at least in part, in the high structural heterogeneity of the conformers that bridge between the active and inactive forms. Such structural diversity, for instance, limits *a priori* the scope of general methods, such as elastic networks and normal mode analysis, which can otherwise be profitably used to identify low-energy collective modes from near-native fluctuations [12, 13].

Here, we introduce and apply a novel comparative tool that can single out those sites that act as hubs in the network of mechanical connections between the receptor residues, i.e. that are crucial for maintaining the integrity of the protein’s large-scale dynamics and mechanics.

We present and discuss this strategy, which is otherwise general and transferable, for the members of a specific GPCR class, namely the class A. This functional group was chosen precisely because of its well-populated and structurally diverse repertoire of conformers.

We analyzed the structural fluctuations across representative conformers to identify those residues that are central for the network of mechanical couplings, and hence the functional dynamics, of the receptors. Such sites have good overlap with known key residues, including those established by purely static structural considerations, but involve additional sites whose functional relevance, that is experimentally verifiable, emerges more clearly from a dynamical perspective.

## Results and discussion

We focus on GPCRs belonging to the rhodopsin-like class A. This class has currently the broadest structural coverage spanning between active, or partially active, and inactive forms. The set includes six different types of receptors, namely: *A*_2*A*_ adenosine, *β*_2_ adrenergic, *M*_2_ muscarinic, *μ*-opioid, neurotensin NTS1 and rhodopsin (see Table 1).

**Table 1:** Structural dataset.

receptor	PDB ID	state	organism	receptor	PDB ID	state	organism
*A*_2*A*_ adenosine	3EML	inactive	H. sapiens	*μ*-opioid	4DKL	inactive	M. musculus
	3PWH	inactive	H. sapiens		5C1M	active	M. musculus
	3REY	inactive	H. sapiens	neurotens. NTS1	4BUO	inactive	R. norvegicus
	3RFM	inactive	H. sapiens		4BV0	inactive	R. norvegicus
	3UZA	inactive	H. sapiens		4BWB	inactive	R. norvegicus
	3UZC	inactive	H. sapiens		4GRV	active (?)	R. norvegicus
	3VG9	inactive	H. sapiens		4XEE	active	R. norvegicus
	3VGA	inactive	H. sapiens		4XES	active	R. norvegicus
	4EIY	inactive	H. sapiens	rhodopsin	1F88	inactive	B. taurus
	2YDO	p. active	H. sapiens		1GZM	inactive	B. taurus
	2YDV	p. active	H. sapiens		1HZX	inactive	B. taurus
	3QAK	active	H. sapiens		1L9H	inactive	B. taurus
	4UG2	active	H. sapiens		1U19	inactive	B. taurus
	4UHR	active	H. sapiens		2G87	inactive	B. taurus
*β*_2_ adrenergic	2RH1	inactive	H. sapiens		2I35	inactive	B. taurus
	3D4S	inactive	H. sapiens		2I36	inactive	B. taurus
	3PDS	inactive	H. sapiens		2J4Y	inactive	B. taurus
	3NY8	inactive	H. sapiens		2PED	inactive	B. taurus
	3NY9	inactive	H. sapiens		3C9L	inactive	B. taurus
	3NYA	inactive	H. sapiens		3C9M	inactive	B. taurus
	3P0G	active	H. sapiens		3OAX	inactive	B. taurus
	3SN6	active	H. sapiens		2HPY	inactive	B. taurus
	4LDE	active	H. sapiens		2I37	inactive	B. taurus
	4LDL	active	H. sapiens		3CAP	active	B. taurus
	4LDO	active	H. sapiens		3PXO	active	B. taurus
	4QKX	active	H. sapiens		2X72	active	B. taurus
*M*_2_ muscarinic	3UON	inactive	H. sapiens		3DQB	active	B. taurus
	4MQS	active	H. sapiens		3PQR	active	B. taurus
	4MQT	active	H. sapiens		4A4M	active	B. taurus

List of PDB entries of the six receptors considered for the bridging score profiling.

### Identifying the mechanical hubs

**Fig 1:**
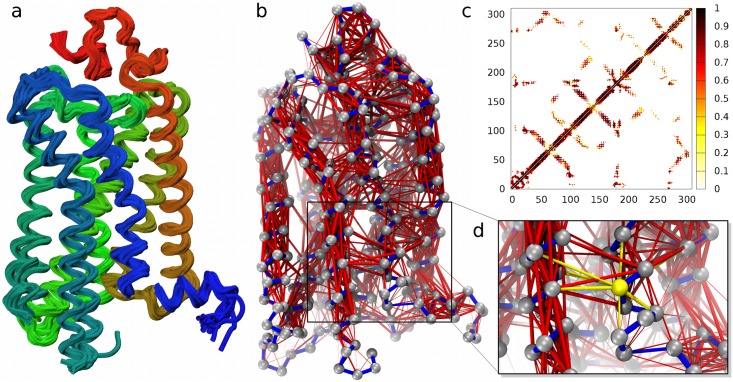
Scanning GPCRs’ mechanical network for key sites. The structural ensemble of a G protein-coupled receptor, see panel (a) for rhodopsin, is used to compute the distance variations for all pairs of amino acids. (b) The pairings in the local mechanical network (C_*α*_ distance <12Å) are highlighted with red bonds with thickness proportional to the observed rigidity; only the strongest links are shown here, while the full network is shown in Fig A in S1 Supporting Information. The network is represented as a color-coded contact map in panel (c). Key residues for the overall mechanical integrity of the network are identified by measuring how the network connectedness varies when one removes all the links of a node corresponding to non-covalent bonds (highlighted in yellow in panel d).

The mechanical hubs of these receptors were identified with a three-step strategy described below and sketched in Fig 1, see Methods for further details.

As a first step, for each receptor we first retrieved all available PDB structures covering its conformational repertoire (Fig 1a). Next, for each pair of residues in spatial proximity (within 12Å on average), we computed their distance variations over the structural set. The variation amplitude is a measure of rigidity, and the residues’ pairwise distance variance can be used as an inverse measure of residues mechanical couplings [14–19]. Hence, this step allows to define the local mechanical network that underpins the receptors functional dynamics (Fig 1b and 1c).

In the final step, each amino acid is profiled based on how much its virtual “mutation”, performed by deleting from the network its local mechanical interactions, changes the network’s connectivity, an approach similar and alternative to measuring the centrality of a particular node in a network (Fig 1d). The higher is the perturbation induced on the network, the higher is the dynamical impact of the considered amino acid. The returned quantity is a measure of the relevance of each residue in establishing indirect couplings between structural fluctuations across distant parts of the receptors. For this reason we shall refer to it as the “mechanical bridging score”.

As we shall discuss later, amino acids with high mechanical bridging score are typically located at the hinge or interface regions between quasi-rigid protein domains and are accordingly well-suited to affect the long-range propagation of structural fluctuations, including functionally-oriented ones. Note that, because we consider intrinsically dynamical properties (structural fluctuations), our notion of bridging score can aptly complement previous GPCRs’ profiling based on networking properties defined from single, static, structures [20, 21].

For a robust identification of the aforementioned mechanical hubs, we combined the six mechanical bridging profiles of the different receptors (Fig B and C in S1 Supporting Information) into a single, average one. The average was taken over the set of corresponding residues (with same GPCRdb numbers [22]) that are shared by all considered receptors. The resulting profile is shown in Fig D in S1 Supporting Information along with its estimated error, which is significantly smaller than the profile variations.

**Fig 2:**
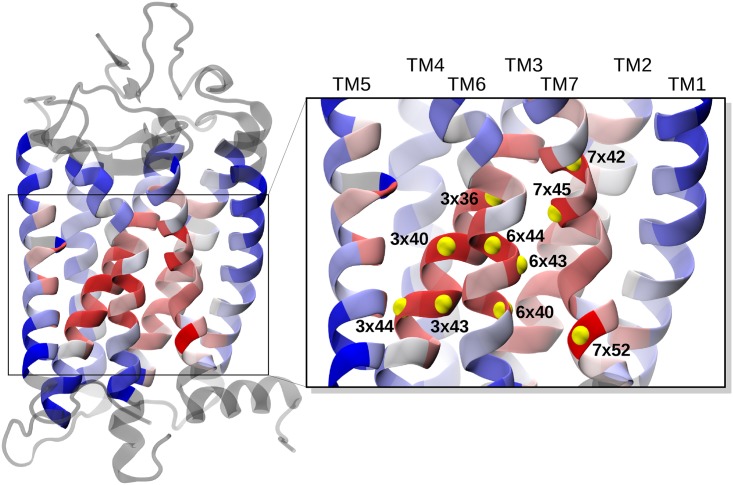
Color-coded profile of the average bridging score. Amino acids in a reference GPCR structure (rhodopsin, PDB ID: 1F88) are color-coded according to the mechanical bridging score averaged over all receptors (blue to red from low to high scores). Residues shown in grey are those with no equivalent positions across the receptors’ ensemble. The top ten ranking sites, listed in the first column of Table 2, are labelled and highlighted with yellow beads in the inset.

The structure of rhodopsin, color-coded according to the average profile, is shown in Fig 2. One can see that the highest average bridging scores are found at the interface between transmembrane helices that are known to be relevant for the receptor activation, namely: TM3, TM6 and TM7 [7, 23]. Note that, compared to these helices, TM4 appears to be much less involved in the large-scale conformational variations of the receptors (see also Fig D in S1 Supporting Information).

### Validating the mechanics-based profiling

The functional relevance of sites with high average bridging score can be shown more stringently by cross-referencing them with the list of currently known key residues for class A receptors based on the survey of Tehan *et al*. [23]. This list of residues was recently compiled by combining sequence- and structure-based selection criteria, that is by singling out residues that are both highly conserved as well as located along the pathway that structurally connects the orthosteric site and the G protein docking site. This connecting region coincides with a hydrophobic core that is central to the helix bundle. The top ranking sites for the average bridging score and those reported in ref. [23] are given in Table 2.

**Table 2:** Key mechanical and functional sites.

top sites for av. bridging score	key functional sites (Tehan et al. [23])	
7x52	1x50	**6x40**
**3x40**	2x46	6x41
7x42	2x50	**6x44**
**6x44**	**3x40**	6x48
7x45	**3x43**	6x50
**3x43**	3x50	7x49
3x36	4x50	7x50
3x44	5x50	7x53
6x43	5x58	
**6x40**	6x30	

The first column provides the ranked list of sites with the highest mechanical bridging score averaged over all receptors of class A. The list of known key functional sites for the same class is shown in the second column. Residues present in both lists are highlighted in boldface. The list of top-scoring residues for each receptor is given in Table A in S1 Supporting Information.

**Fig 3:**
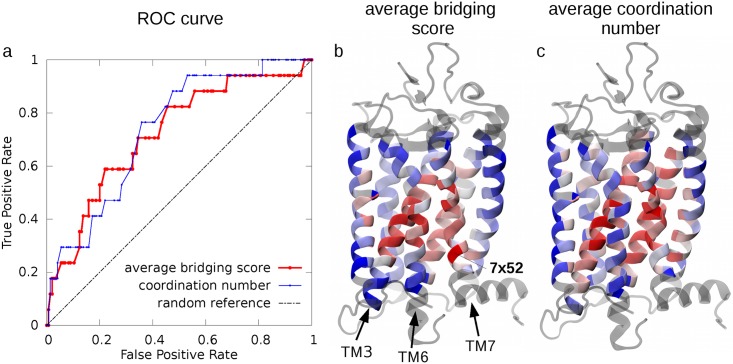
Functional profiling of key sites for GPCR’s mechanical and structural networks. (a) The list of known GPCRs functional sites in Table 2 is used for the ROC curve profiling of the top mechanical sites in Table 2 (red) and of those that have the highest structural coordination (number of contacts) across the receptor ensemble (blue). For reference, the performance of a random classifier is shown by the dashed black line. Color-coded representations of the average bridging score and of the average coordination number are shown for rhodopsin (PDB ID: 1F88) in panels (b) and (c), respectively. The representation in panel (b) is the same as in Fig 2. The coordination number averaged over the six receptors shown in panel (c) ranges from 18.7 (blue) to 47.4 (red).

The overlap between our top ranking sites and the known key functional residues reported by Tehan *et al*. [23] was assessed by using the receiver operating characteristic (ROC) curve in Fig 3a. The curve shows that by running through our ranked list of residues, the “discovery” of the known functional sites occurs at a significantly higher rate than expected for a random reference case (the plot diagonal).

This is an indication that the average bridging score is able to capture with a significant degree of sensitivity those residues likely to be involved in the functionality of class A GPCRs.

This conclusion is further supported by comparing the ranking based on the average bridging score with one based on a purely static structural criterion. To this end, we ranked the amino acids based on their number of contacts. This allows for a transparent and equal-footing comparison, since the criterion exclusively considers the average amino acid connectedness, regardless of whether a contact is associated to a strong (i.e. rigid-like) mechanical coupling or not. This structure-based ranking criterion is inspired by previous works on GPCRs [20, 21] that demonstrated a correlation between sites with functional relevance and graph properties of the static contact map build on single receptor structures. This is confirmed by the marked deviation of the corresponding ROC curve from the diagonal in the plot of Fig 3a. The key observation that is relevant here is that the average bridging score ROC curve is well in line with the structure-based one, thus underscoring the functional significance of the mechanics-based ranking criterion. In addition, it prompts to understand the different insight that it can offer over pure structural approaches.

To clarify the latter point, we show in Fig 3b and 3c and Fig D in S1 Supporting Information the profiling of residues according to the dynamical or structural criteria. The comparative inspection indicates that the differences are mostly localized at specific portions of TM6 and TM7, which are high ranking for the mechanical bridging score, but not for the structural one. These regions, therefore, appear to have a key role across class A members that is genuinely tied to the receptors’ functional mechanics and hence cannot be detected from static structural observables.

### Functional role of key mechanical hubs

The 10 sites with the highest average bridging score (Table 2) include residues forming the so-called hydrophobic hindering mechanism (HHM: 6x44, 3x43 and 6x40). Mutagenesis experiments have shown that this conserved hydrophobic triplet, that is contacted by other listed residues, namely 3x40, 6x43 and 3x44, is essential for the activation process of class A GPCRs [23]. The HHM triplet plus the proximal site 3x40, which has the second highest score, all take part in the structural rearrangements bridging the inactive and active state. The latter, in fact, depends on the HHM opening for establishing the water channel in the active conformation [23]. Residue 3x40 additionally participates in the transmission switch [7] and is highly conserved as a branched hydrophobic residue as well, see Table A in S2 Supporting Information [23].

Residue 7x42 is, instead, involved in a different molecular switch, i.e. the TM3-TM7 lock [7]. This is the main mechanism responsible for activation in rhodopsin and possibly one of the first switches triggered by ligand binding in other GPCRs. Position 7x45 is one of the most conserved residue in TM7 (Table A in S2 Supporting Information) [7]. Finally, the 3x36 position, though not conserved, was shown by site-directed mutagenesis experiments to have a stabilizing role for the inactive state [7].

Most of the top scoring residues listed in Table 2 are therefore sites with a demonstrated involvement in class A GPCRs activity. This validates the viability of dynamical profiling approaches in general, and the mechanical bridging score in particular, for singling out functionally important residues and providing a rationale for their relevance. Given these premises, of particular interest are those sites that have a high bridging score, but are not yet known as functionally relevant.

This is the case for site 7x52, that has the highest score in our analysis. This amino acid is part of the well-conserved motif NPxxY(x)_5,6_F, but is otherwise not particularly central in the static network of contacts, see Fig 3c and Fig D in S1 Supporting Information. Its functional relevance therefore has not been fully investigated before, though its possible participation in stabilising the TM6–TM7 interhelical interaction has been suggested by [24]. Mutations at position 7x52 were shown to constitutively activate the TSH (thyroid stimulating hormone) receptor [5, 25] by possibly disrupting the packing between TM6 and TM7. We therefore suggest site 7x52 as a putative novel site crucial for functionality. Again, the fact that its relevance does not emerge from structural considerations indicates that its role is likely to be a genuinely dynamical, or mechanical one.

We finally note that the highest scoring sites in Fig 2 are immediately adjacent to the region that the latest studies of refs. [26, 27] have identified as the most structurally affected by the activation/inactivation transitions. In particular, by comparing class A GPCRs with different activation states, Venkatakrishnan *et al*. [27] identified three G protein-coupling residues, 3x46, 6x37 and 7x53, whose contacts are disrupted during activation, and that are exposed to the G protein-binding pocket by the dislocation of the cytoplasmic side of TM6 away from the helix bundle.

A comprehensive and annotated list of sites so far addressed in mutagenesis experiments of class A GPCRs is provided in Table B in S2 Supporting Information. Further mutagenesis probings of residue 7x52, though for non-class A GPCRs, are given in Table C in S2 Supporting Information. The data in Table B and C, while not necessarily transferrable to a different class, are still fully consistent with our conclusion that site 7x52 has a key functional role and ought to be a good candidate for future mutagenesis experiments.

This conclusion is further supported from the bioinformatics analysis of the degree of evolutionary conservation of the key residues identified in this study (Table A in S2 Supporting Information). In particular, the physico-chemical characteristics of the residue in position 7x52 are highly conserved in all class A GPCRs from eukariotes. Specifically, in more than 80% of the sequences, the corresponding amino acid is branched and hydrophobic. This underscores the functional relevance of this position from an evolutionary point of view. Similar conservation trends are found for other residues of Table A in S2 Supporting Information, that are key for the functional mechanics, particularly the activation, of the receptors.

### Analysis of *μ*-opioid receptor MD simulation and receptors’ rigid domains

The conclusions of the previous section are supported by two complementary extensions of the analysis above. Specifically, we first repeated the bottom-up mechanical profiling of residues for a single receptor using an ensemble of structures obtained from a molecular dynamics simulation. Finally, we examined the mechanical role of residue 7x52 by using a top-down approach based on the quasi-rigid domain decomposition of all receptors.

For the first extension, we applied our protocol to conformers sampled by extensive atomistic molecular dynamics (MD) simulations of the *μ*-opioid receptor [28] started from both the inactive state and the ligand-bound active one. The MD ensemble provides a richer sampling of the active and inactive conformers and hence allows to capture the internal dynamics and mechanics with greater fidelity than from the sole pair of available crystal structures.

**Fig 4:**
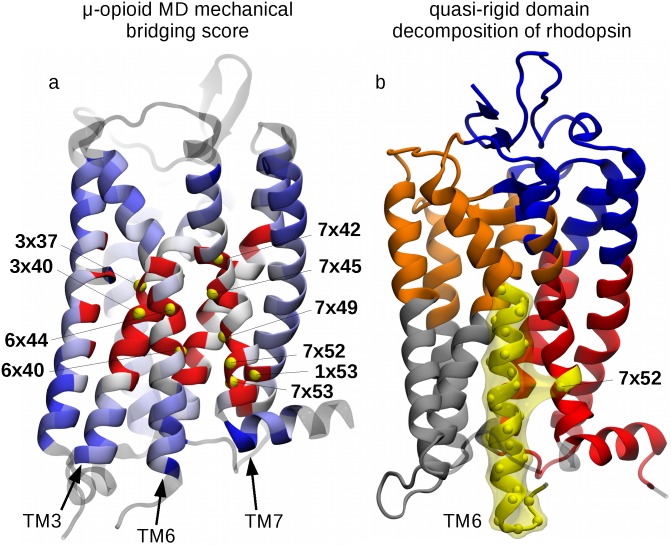
Functional role of site 7x52: MD simulations and quasi-rigid domain decomposition. (a) Amino acids of the *μ*-opioid receptor (PDB ID: 4DKL) are color-coded according to the mechanical bridging score computed from atomistic molecular dynamics simulations. The color convention is the same as in Fig 2, with the top 10 ranking residues being labelled and highlighted with yellow beads, corresponding to the following sites, in decreasing order of score: **6x40**, 7x52, 7x45, **3x40**, 1x53, **7x49**, 7x42, **7x53**, 3x37, **6x44** (in boldface, the key functional sites also present in the list of Tehan *et al*. [23]). Panel (b) shows the optimal SPECTRUS [19] decomposition of rhodopsin into 5 quasi-rigid domains. The TM6-based domain is highlighted in yellow and it notably includes residue 7x52 from TM7. Analogous decompositions for the other receptors are shown in Fig I in S1 Supporting Information.

The results of the single-residue analysis for the *μ*-opioid receptor (Fig 4a) are well consistent with those of Fig 2, based on the cumulated profiles of all six receptors. Specifically, the highest scoring residues, highlighted in Fig 4a and listed in the caption, include conserved residues of helices TM3, TM6 and TM7, two residues of the HHM (6x40 and 6x44) and, again, site 7x52.

The analysis of the *μ*-opioid receptor MD simulation helps clarify a further important question, that is how sensitive is the bridging score profile to the size of the conformational ensemble. To this end, we measured the Pearson correlation coefficients between the profile computed from the combined active and inactive MD trajectories and the profile obtained from the two available experimental structures for *μ*-opioid receptor, corresponding to its active and inactive forms. In spite of the very different size of the two datasets, the profiles, shown in Fig E in S1 Supporting Information, are remarkably similar and their Pearson correlation coefficient is as high as 0.80.

A similar analysis has been performed on additional MD simulations run for M_2_ muscarinic receptor, including a 190ns-long simulation of the inactive state (PDB ID: 3UON) and a 200ns-long one for the active state (PDB ID: 4MQS) (for more details about the MD simulations setup, see the relative section in S2 Supporting Information). The resulting comparison is reported in Fig E in S1 Supporting Information as well, and again a very high correlation (0.87) with the original score based on crystal structures has been observed.

More insight into this result is provided by the additional analysis reported in Fig F in S1 Supporting Information which conveys, in the form of a color-coded matrix, the Pearson correlation coefficients between the profile computed from the combined trajectories and the profile computed from various pairs of snapshots picked at various points of either or both simulations. The matrix vividly shows that, despite the dataset size differences, the consistency of the profiles can be very high as long as the two snapshots are diverse enough to represent both the active and inactive forms.

Analogous conclusions hold for the other five receptors, see Fig G in S1 Supporting Information, for which an equally meaningful bridging score could be derived based solely on a single pair of active-inactive conformations. As an immediate consequence of this fact, the comparison between the score profiles from all the possible pairs of active-inactive structures of a receptor allowed us to assign error-bars to each data point, which are consistently smaller than the local profile variations we are interested in measuring (see Fig H in S1 Supporting Information).

We finally turn to the top-down analysis based on the quasi-rigid domain decomposition of the six class A receptors. To this purpose we used the SPECTRUS webserver [19]. This performs an optimal domain decomposition based on the internal distance variations across a set of representative structures. The analysis, an example of which is illustrated in Fig 4b for rhodopsin, presented two salient features that recurred across the different receptors.

First, the intracellular half of TM helix 6 was systematically identified as a quasi-rigid domain, consistent with its role in the internal rearrangements accompanying the receptors’ activation [23].

The second feature is that residue 7x52 is often assigned to the same rigid domain as TM6. Such domain association is interesting because intuitively one would otherwise always assign 7x52 to the TM7-based domain, to which it structurally belongs, see Fig 4b. As a matter of fact, site 7x52 is recognised part of the TM6 dynamical domain in a sizeable fraction (∼25%) of the subdivisions from 2 to 10 domains of the receptors, including the *μ*-opioid receptor MD simulations, see Fig I in S1 Supporting Information. This means that the displacements of 7x52, unlike other sites in TM7, are appreciably coupled with those of the cognate helix, TM6. Accordingly, 7x52 appears to act as an interface, bridging site between the two distinct mobile TM6- and TM7-based domains, as it is illustrated in Fig 4b for rhodopsin.

The recurrent difference of the dynamics- and structure-based assignment is consistent with the other evidence presented above that residue 7x52, whose functional role is still largely unexplored, is likely relevant for the mechanical response of class A GPCRs.

### Concluding remarks

The current understanding of GPCRs functionality, and primarily the response to ligand binding, has been significantly shaped by the analysis of the growing number of their structures solved with X-ray or NMR [29]. Though such structures give valuable clues for the active states of GPCRs, they still include a limited set of snapshots of the likely conformational states induced by agonist and G protein binding. In addition, both experiments and atomistic MD simulations indicate that the receptors are capable of adopting multiple conformations, depending on the nature of the bound ligand. Our insight into the agonist- and G protein-initiated conformational changes is therefore still limited.

As a step towards clarifying this open problem, we devised and applied a strategy for identifying key sites presiding the functional dynamics and mechanics of class A GPCRs. This is the largest subclass and it has arguably the widest structural coverage, with conformers from 6 different receptor types (including rhodopsin) in different activation states. We analysed the internal structural fluctuations across the dataset. In particular, we focussed on the pairwise distance variations of corresponding amino acids which were used to infer the network of local mechanical couplings that underpin the large scale, and arguably functionally-oriented conformational changes. The mechanical network was finally analyzed to locate the few sites that most contribute to GPCR’s collective mechanics. To do so we identified the residues whose virtual deletion leads to the strongest softening of the overall mechanical response.

The viability of the approach to single out the most relevant functional sites was validated by the significant overlap between key sites for mechanical response and those known to be crucial for function based on independent and different criteria.

On the one hand, this result provides a concrete and vivid illustration of the relevance of dynamics- and mechanics-based criteria for locating key sites for enzyme functionality and hence prompts their use in combination with other more established structure-based static criteria.

On the other hand, the validation revealed that mechanically-relevant sites at interface between transmembrane helices 6 and 7 were not included in the list of previously known functionally-relevant positions. This was particularly the case for site 7x52, which is among the highest ranking ones for the mechanical response, and whose relevance is supported by the analysis of both atomistic MD simulations of the *μ*-opioid receptor as well as the analysis of GPCR’s rigid-domain decompositions.

Based on these convergent indications, we conclude that site 7x52 likely plays a key role in the conformational dynamics of class A GPCRs. Its functional relevance, as well as that of other sites in the central region of the transmembrane helical bundle, ought to be experimentally verifiable, e.g. with site-directed mutagenesis experiments.

## Methods

### Network of dynamical similarities

The receptors’ mechanical network was inferred from the analysis of distance variations between pairs of amino acids. These, in fact, are key elements to define the subparts of the proteins that interact in such a concerted manner that they behave as quasi-rigid domains [19]. The distance variation *f*_*a*,*b*_ between two residues *a* and *b* is computed as the standard deviation of the distances *d*_*a*,*b*_ between their C_*α*_ atoms over two or more structures (PDB entries or snapshots from MD simulations):

$$f_{a,b}=\sqrt{\left\langle d_{a,b}^{2}\right\rangle -{\left\langle d_{a,b}\right\rangle }^{2}}.$$ (1)

The strength (rigidity) of the pairwise mechanical couplings is then quantified with a Gaussian weighting of the corresponding distance variations

$$\sigma_{a,b}=\exp\left(-f_{a,b}^{2}/2{\bar{f}}^{2}\right),$$ (2)

Because we are interested to define the receptors’ mechanical network in terms of physical, local coupling between amino acids, we set *σ*_*a*,*b*_ = 0 for amino acids whose C_*α*_’s are at an average distance larger than 12Å, see Fig J in S1 Supporting Information. The value of the sensitivity parameter, $\bar{f}$, in Eq 2 is then set as the average of *f*_*a*,*b*_ over the amino acids pairs closer than 12Å.

### Mechanical bridging score

To define the key mechanical bridging sites, or hubs, of the receptors, we resort to the spectral clustering analysis of the mechanical network [30, 31].

Specifically, given the matrix, *σ*, of couplings between *N* amino acids, we characterize the spectrum of the symmetric Laplacian matrix,

$$L=I-D^{-1/2}\;\sigma \;D^{-1/2},$$ (3)

where *I* is the identity matrix and *D* is the degree matrix *D*_*a*,*b*_ = *δ*_*a*,*b*_ ∑_*c*_ *σ*_*a*,*c*_. Its non-negative eigenvalues 0 = *λ*_0_ ≤ … ≤ *λ*_*i*_ ≤ … ≤ *λ*_*N*−1_ provide information about how well the network is neatly partitioned in distinct clusters (mechanical domains) and, accordingly, are typically used to define optimal subdivisions of the network.

Here, the eigenvalues will be used for a different goal, namely to ascertain how important is each node to maintain the overall mechanical connectedness of the network. This amounts to measuring how much the network Laplacian spectrum changes when the connections, or couplings, of a node with its neighbors (excluding the connections corresponding to bonded interactions) are deleted. This response for residue *k* is given by the mechanical bridging score:

$$\Delta_{k}=\Omega_{k}-\Omega^{0}.$$ (4)

where $\Omega^{0}={\tilde{\sum }}_{i=1}^{N-1}\frac{1}{\lambda_{i}}$ is the sum of the inverse eigenvalues (the tilde superscript denotes the omission of zero eigenvalues) for the full network, and *Ω*_*k*_ is the same quantity but calculated for the network where the couplings relative to the *k*th node have been deleted.

The bridging score profile is computed separately for each receptor using its available structural representatives. The average bridging score is then obtained by averaging the bridging score over all equivalent positions of the various receptors.

### Class A GPCRs database

The structures used for the analysis are listed in Table 1. Among the receptors whose structure is reported in the Protein Data Bank, we selected those for which both active and inactive conformations were known. These include the following receptors: *A*_2*A*_ adenosine, *β*_2_ adrenergic, *M*_2_ muscarinic, *μ*-opioid, neurotensin NTS1, rhodopsin. Moreover, we applied the same analysis on an MD trajectory as well, obtained by merging two simulations of the *μ*-opioid receptor [28], starting from the inactive state (PDB ID: 4DKL [32]) and the active state bound to the agonist BU72 (PDB ID: 5C1M [33]).

Each of the six receptors included in our dataset had a minimum of two crystal structures (*μ*-opioid receptor) and a maximum of 21 (rhodopsin), including both active and inactive conformations.

The GPCRdb numbering scheme [22] has been used to match the residue positions common to all receptors. This scheme consists of the combination of two numbers in the form AxBB, where the first one is the helix number, while the second one is a progressive number chosen so that the most conserved residue in each helix has the value of 50.

Note that, because our main goal is to identify the key residues that are common across the various GPCR types, the analysis must necessarily focus on those amino acids that are in one-to-one correspondence across the heterogeneous GPCR set. This requirement lead, *de facto*, to exclude the residues involved in EL/IL loops, though one should be aware that their role in receptors’ activation is increasingly acknowledged [34]. Likewise, when defining the set of common positions, those residues, close to the intra- and inter-cellular regions, for which the process of cutting the surrounding connections could lead to unwanted disconnections of the network, were not included. Consequently, the remaining set of positions correspond to the transmembrane region of the receptors, with numbering: 1x36–1x56, 2x40–2x63, 3x24–3x54, 4x42–4x61, 5x38–5x60, 6x34–6x57, 7x36–7x43, 7x45–7x55.

## Supporting information

S1 Supporting Information Additional figures. (PDF)

S2 Supporting Information Additional tables and MD setup descriptions. (PDF)

S1 Additional Data Annotated PDB files. Contains all PDB structures used for the analysis, annotated with the GPCRdb numbering scheme. For each receptor, a decomposition into quasi-rigid domains is provided, along with the corresponding single-receptor bridging score.
(ZIP)
