# Supplementary material for: Unifying view of mechanical and functional hotspots across class A GPCRs
Source: PLoS Comput Biol. 2017 Feb 3;13(2):e1005381. doi: 10.1371/journal.pcbi.1005381 (PMC5315405; doi:10.1371/journal.pcbi.1005381)
Supplement: S1 Supporting Information — (PDF) [file pcbi.1005381.s001.pdf]

S1 Supporting Information

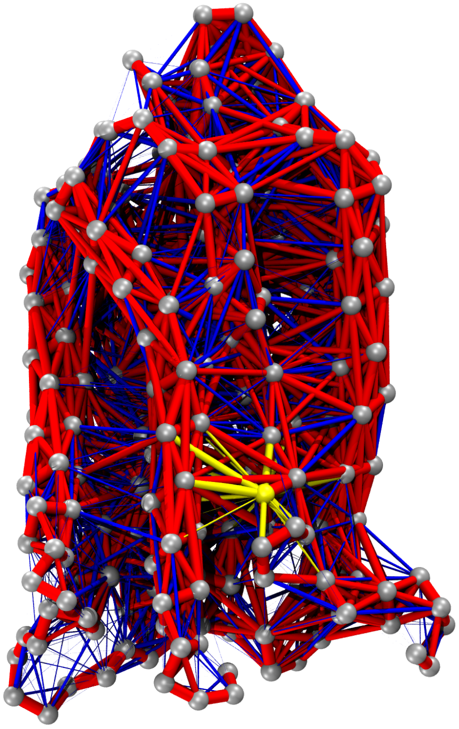

Figure A: **Mechanical network of rhodopsin.** The full mechanical network for rhodopsin is shown here, connecting all C_α's whose distance is less than < 12Å on average. The strongest links are colored in red, while the weakest ones are in blue. For residue 7x52, the links corresponding to non-covalent bonds are highlighted in yellow.

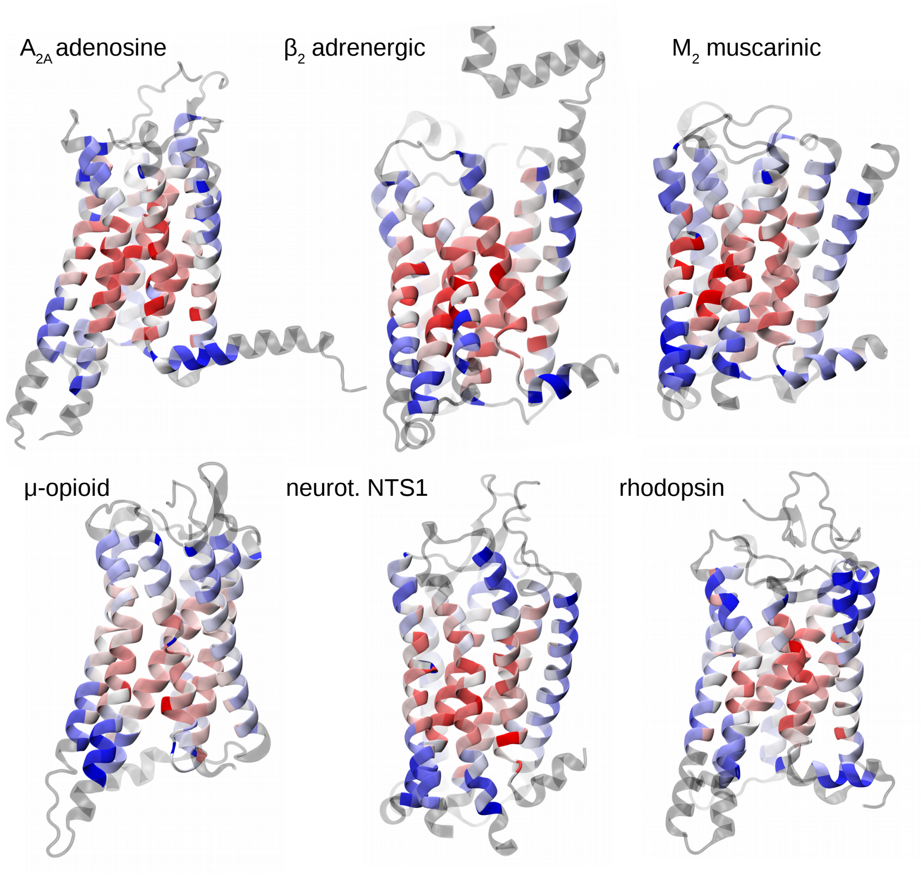

Figure B: **Mechanical bridging score for the six GPCRs considered in this work.** The score is presented with a decreasing color scale from red to blue, on one of their conformations (PDB IDs: 4UHR, 4QKX, 4MQT, 5C1M, 4XES, 4A4M). The structures are oriented with TM6 and TM7 up front.

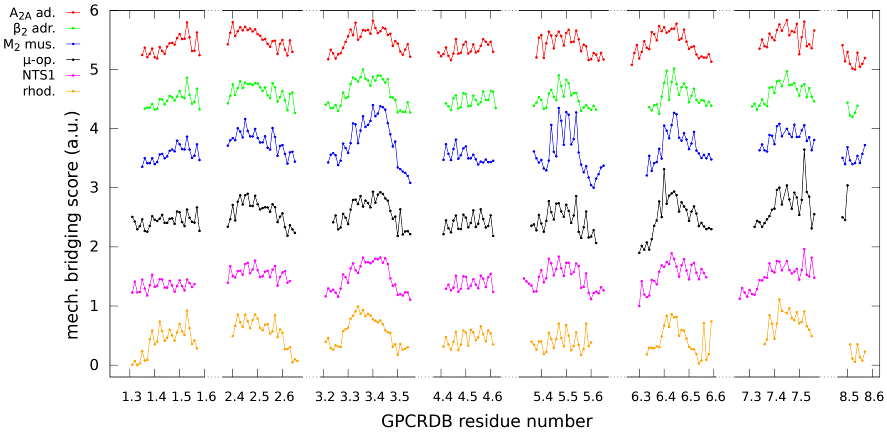

Figure C: **Sequence profiles of the mechanical bridging score.** The residues are numbered according to the GPCRdb scheme.

A_{2A} aden.	β_2 adren.	M_2 musc.	M_2 m.(MD)	μ-opioid	μ-op.(MD)	neur.NTS1	rhodopsin
**7.45**	**6.44**	**3.4**	**3.4**	**7.52**	**6.4**	**7.52**	**7.42**
**3.4**	**3.36**	**3.43**	5.47	**6.4**	**7.52**	**6.43**	3.34
**7.52**	6.41	**3.44**	**3.43**	8.5	**7.45**	5.47	7.46
2.4	**7.45**	5.47	5.54	**7.45**	**3.4**	**3.43**	**3.36**
1.53	5.47	3.45	**3.44**	**7.42**	1.53	**3.36**	1.53
3.33	3.42	5.5	3.37	7.41	7.49	7.55	7.43
6.45	**3.43**	5.54	**3.36**	**6.44**	**7.42**	3.45	**7.52**
7.49	**3.4**	**6.44**	3.39	**3.4**	7.53	5.43	3.33
**7.42**	3.35	3.42	2.49	7.53	3.37	3.41	7.49
6.38	3.32	6.45	6.48	**3.43**	**6.44**	**6.44**	**7.45**

Table A: **Top-scoring residues for the six class A GPCRs.** The key positions according to the average bridging score (see Table 2), obtained by combining the six single-receptor profiles, are highlighted in boldface. The list includes the top ranking residues from the analysis of μ-opioid and M_2 muscarinic MD simulations as well.

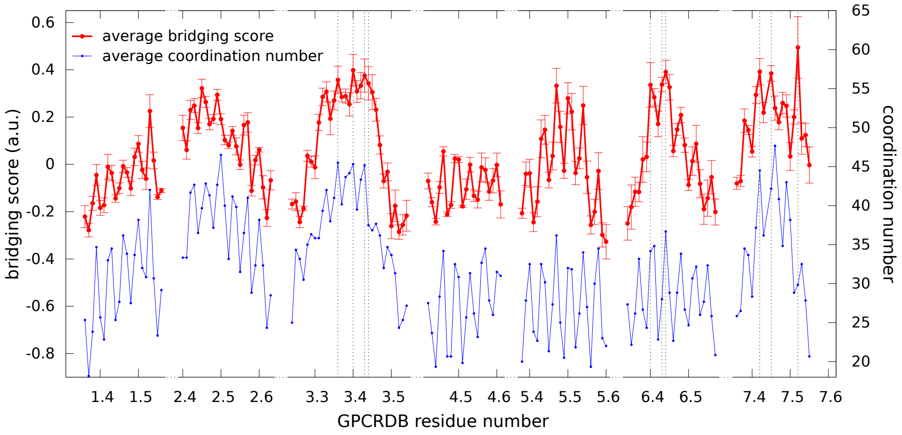

Figure D: **Profiles of the average bridging score (red) and coordination number (blue).** The averages are taken over the six considered receptors. Note that the bridging score profiles are defined up to an additive constant. For the purpose of computing the average, the additive constant for each receptor was set so that the mean bridging score computed over all its residues is equal to zero. The error bars represent the standard error of the mean. The positions of the 10 top ranking residues of the bridging score, as listed in the first column of Table 2, are highlighted with the dashed vertical lines.

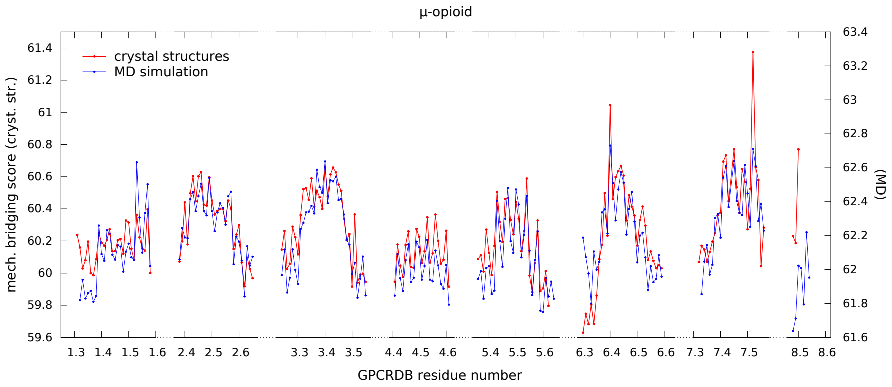

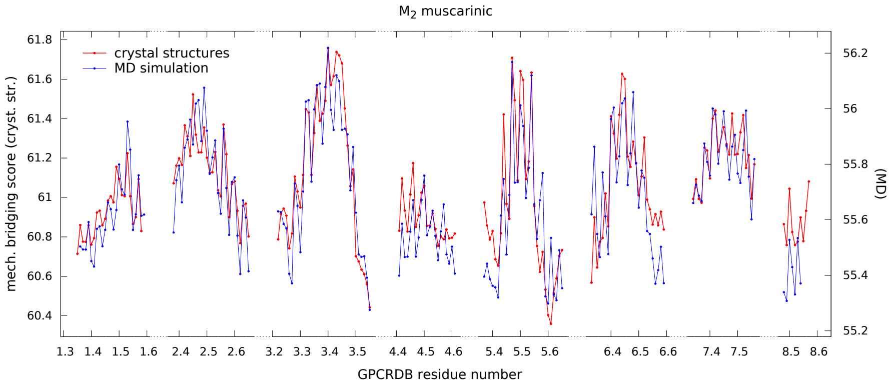

Figure E: **Comparison of bridging score profiles based on PDB structures and MD simulations of the μ-opioid and M_2 muscarinic receptors.** In both figures, the blue curve is the profile based on the combined MD simulations of the active and inactive forms. The red profile is computed from the available experimental structures of the active and inactive forms (two structures for μ-opioid receptor and three for M_2 muscarinic receptor). In both cases, the two profiles are very similar and significantly correlated. Their Pearson correlation coefficient is, respectively, 0.80 and 0.87, while the Kendall nonparametric correlation test gives a z-score beyond 13 (p-value $< 10^{-40}$) for μ-opioid receptor, and beyond 14 (p-value $< 10^{-45}$) for muscarinic receptor.

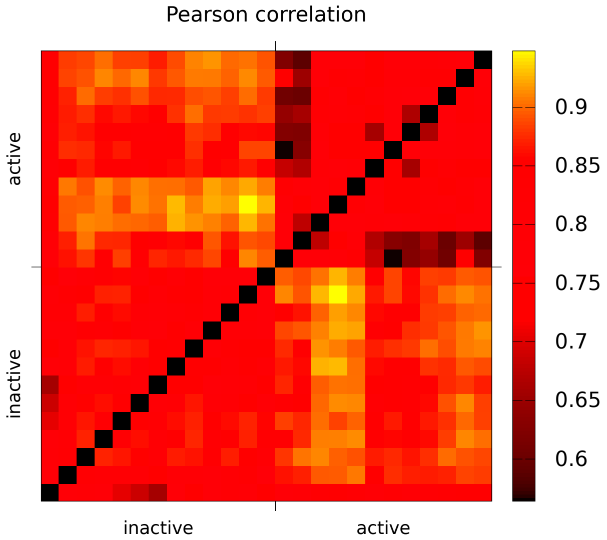

Figure F: **Robustness of the bridging score profile of the μ-opioid receptor computed from MD simulations.** We compared the profile computed from the entire structural dataset of the μ-opioid receptor sampled in the combined MD trajectories started from its active and inactive forms, and compared it with the profile computed using two sole snapshot from either or both trajectories. The consistency was measured with the Pearson correlation coefficient computed between the whole-ensemble profile and the various pairs of two snapshots. The results are presented in the form of a color-coded matrix. It is readily seen that the correlation of the profiles computed from the sole set and from as few as few structures are in excellent accord whenever the two structures include snapshots from the active and inactive forms (i.e. when they capture the structural breadth of the biologically relevant conformational ensemble).

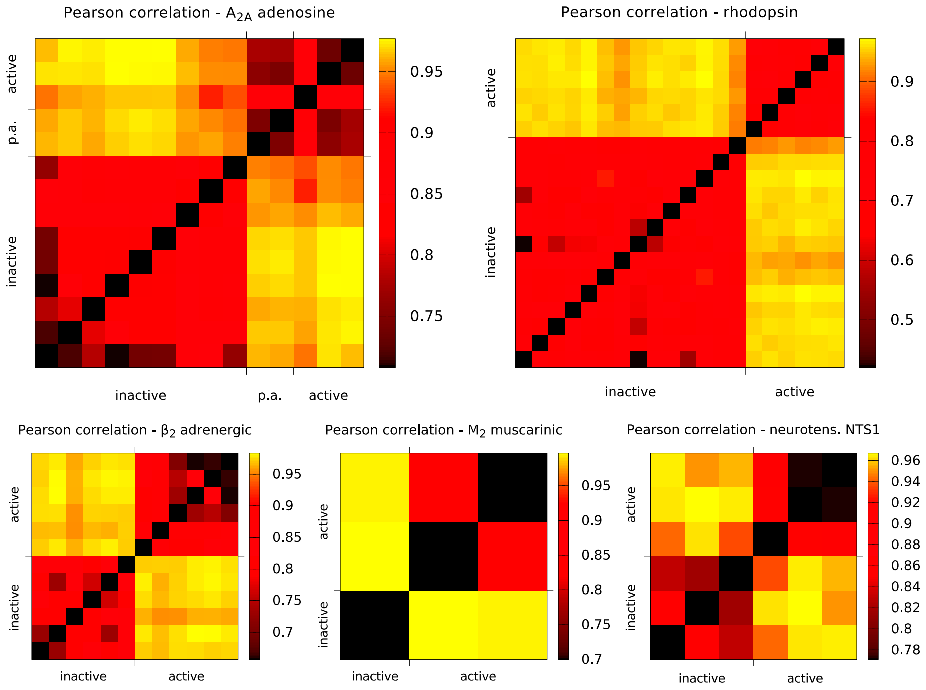

Figure G: **Robustness of the bridging score profile with respect to the dataset size.** For a stringent test of robustness we measured the Pearson correlation coefficient between the profile computed from the entire structural dataset available for a given receptor, and the profiles obtained by considering a single pair of structures in the set. The correlation was computed for all possible pairs of structures and are hence presented for each receptor type (except for μ-opioid receptor, discussed after in Fig. F) in the form of a color-coded matrix. It is readily seen that the correlation of the profiles computed from as few as two structures are in excellent accord whenever the two structures include active and inactive forms (i.e. when they capture the structural breadth of the biologically relevant conformational ensemble).

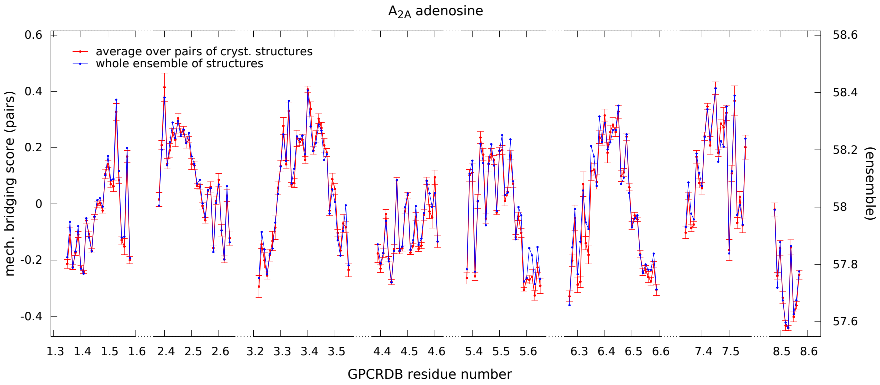
A2A adenosine
average over pairs of cryst. structures
whole ensemble of structures
mech. bridging score (pairs)
(ensemble)
GPCRDB residue number
1.3 1.4 1.5 1.6 2.4 2.5 2.6 3.2 3.3 3.4 3.5 4.4 4.5 4.6 5.4 5.5 5.6 6.3 6.4 6.5 6.6 7.4 7.5 8.5 8.6

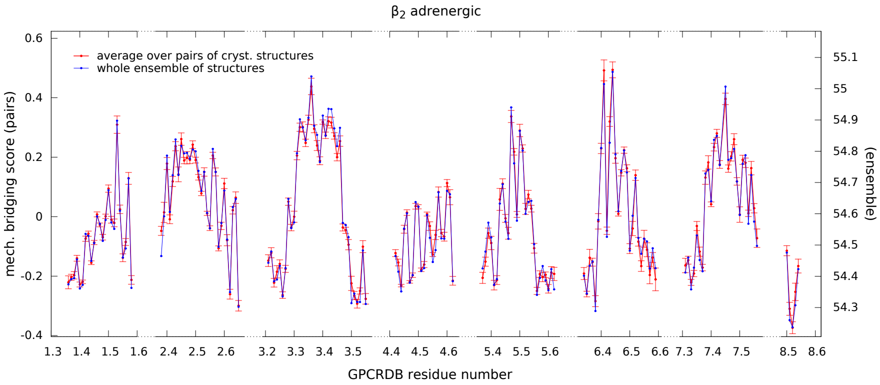
β2 adrenergic
average over pairs of cryst. structures
whole ensemble of structures
mech. bridging score (pairs)
(ensemble)
GPCRDB residue number
1.3 1.4 1.5 1.6 2.4 2.5 2.6 3.2 3.3 3.4 3.5 4.4 4.5 4.6 5.4 5.5 5.6 6.4 6.5 6.6 7.3 7.4 7.5 8.5 8.6

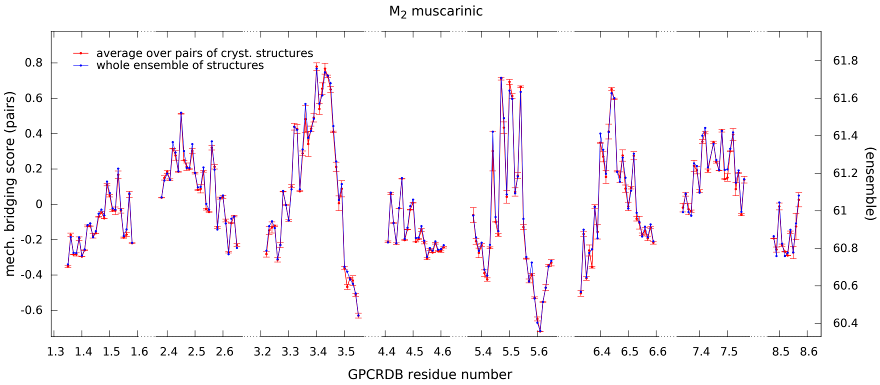
M2 muscarinic
average over pairs of cryst. structures
whole ensemble of structures
mech. bridging score (pairs)
(ensemble)
GPCRDB residue number
1.3 1.4 1.5 1.6 2.4 2.5 2.6 3.2 3.3 3.4 3.5 4.4 4.5 4.6 5.4 5.5 5.6 6.4 6.5 6.6 7.4 7.5 8.5 8.6

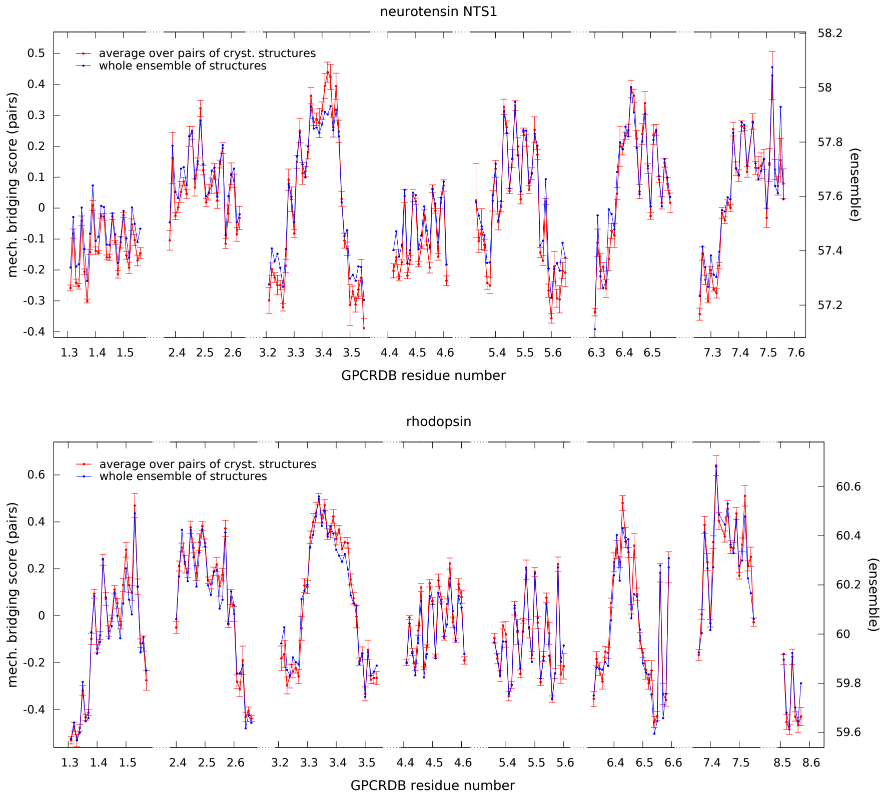

Figure H: **Error analysis for the mechanical bridging score profile.** For a practical, empirical estimate of the statistical uncertainty on the single-receptor profiles, we computed the latter for all possible pairs of inactive and (possibly partially) active structures as labelled in Table 1. After setting the average profile value to zero (see caption of Fig. D), we next computed the average profile over all such pairs and the associated error was conservatively take as the standard deviation on the sample divided by the square root of the active or inactive forms, whichever is smaller. For comparison, the score computed on the whole ensemble is also shown.

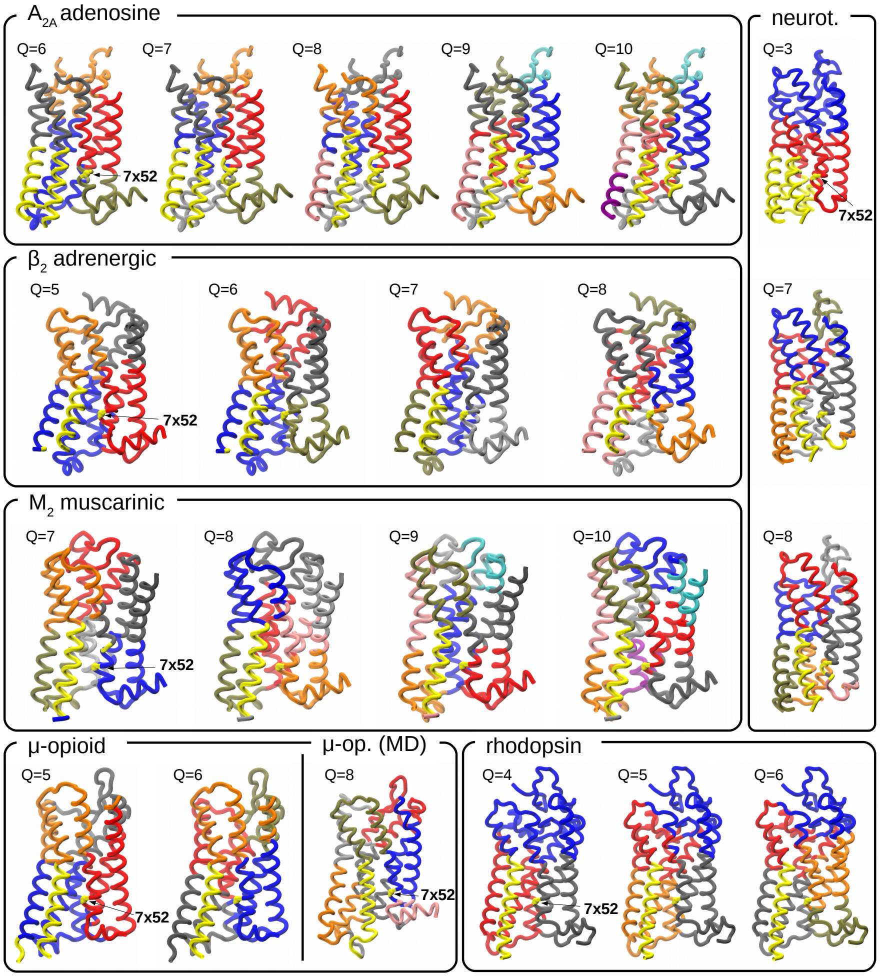

Figure I: **Quasi-rigid domain decompositions of receptors.** The decompositions into quasi-rigid domains of the six GPCRs were produced by the SPECTRUS webserver (http://spectrus.sissa.it). Those decompositions including a TM6+7x52 domain have been selected, and this particular domain is highlighted in yellow. For μ-opioid receptor, a subdivision based on conformations from the MD simulation is shown as well.

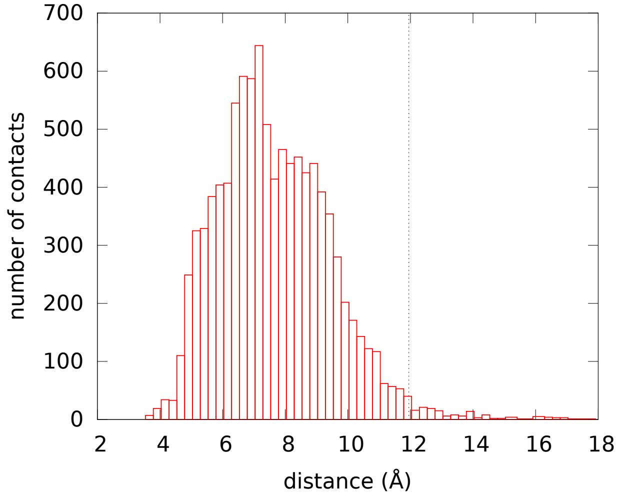

Figure J: **Distribution of interhelix C_α-C_α distances.** Histogram of the distances, for all six receptors considered in our analysis, between each residue's C_α and its nearest neighbor one, belonging to the closest facing helix. A threshold of 12Å, corresponding to the 98.5th percentile (dashed vertical line), guarantees that the great majority of connections is included, without disrupting contacts between helices.
