# Supplementary material for: Unifying view of mechanical and functional hotspots across class A GPCRs
Source: PLoS Comput Biol. 2017 Feb 3;13(2):e1005381. doi: 10.1371/journal.pcbi.1005381 (PMC5315405; doi:10.1371/journal.pcbi.1005381)
Supplement: S2 Supporting Information — (PDF) [file pcbi.1005381.s002.pdf]

Table A: Position conservations (top conserved residue identities and their percentages), for residues discussed in the paper, across eucariotic class A GPCRs, from the curated GPCRdb server alignment (Isberg, 2014)

Generic residue numbering (class A)	Residue Conservation			
**3.36**	M=19%	G,L=12%	V,C=10%	F,S=8%
**3.40**	I=40%	V=24,3%	L=11,5%	A=5%
**3.43**	L=73%	I=10%	V=5%	F,G,H,T=1% each
**3.44**	T=24%	C=16%	A,V,L=12%	
**6.40**	V=35%	I=28%	L=15%	M=6%
**6.43**	V=30%	A=22%	L,I=11%	
**6.44**	F=71%	Y=6%	V=5%	L=3%
**7.42**	A=38%	G=22%	I=7%	S=10%
**7.45**	N=59%	S=15%	H=10%	
**7.52**	I=41%	L=32%	V=13%	Y,C=4% each
**7.53**	Y=85%	F=4%	L=3%	

To allow comparison of residues in equivalent position in both inactive and active receptors state of GPCRs, we used the GPCRdb numbering scheme (Isberg, 2016). To calculate the conservation of the residue analyzed in this article we obtained the multiple alignment from the GPCRdb and defined the percentage of conservation as (X/P)*100 where X is the residue and P the length of the multiple alignment. Plants are not included since GPCR in plants are not well characterized (Taddese, 2014). The alignment of 1618 non-olfactory (the inclusion of curated sequences of about 3000 olfactory receptors does not change the conservation values) class A GPCRs were obtained from the GPCRdb database. A total of 276 positions, spanning all the seven transmembrane segments were aligned.

Table B: Effects of mutations in residues involved in this article within class A GPCRs

Generic residue numbering (class A)	Receptor	Mutants	Effect	Reference
**3.40**	M1 muscarinic	V3.40A	Increased agonist affinity	Hulme (2013)
	M5 muscarinic	V3.40A	Increased agonist affinity	Hulme (2013)
	MOR136-1	S3.40A	Abolished agoinist binding	Ho (2015)
	Histamine H1R	I3.40A	Lower basal activity and lower agonist response	Sansuk (2011)
	Histamine H1R	I3.40G	Lower basal activity and lower agonist response	Sansuk (2011)
	TSHR	V3.40A	Constitutive activity	Duprez (1994)
**3.43**	S1P1	L3.43E/G	Abolished activation	Fujiwara (2007)
	B2AR	L3.43R/K/A	Increased basal activity	Tao (2000)
	M1 muscarinic	L3.43A	Constitutive activity	Lu (1999)
	TSHR	L3.43Q/N/R	Constitutive activity	Kosugi (2000), Nishihihara (2006), Trulzsch (2001)
	C5A	L3.43A	Constitutive activity	Baranski (1999)
	CB1	L3.43A	Constitutive activity ; elevated basal cAMP accumulation ; enhanced affinity for agonists and diminished affinity for inverse agonists.	D'Antona (2006)
**6.40**	C5A	V6.40A	Increased basal activity	Baranski (1999)
	M5 muscarinic	I6.40S	Constitutive activity	Spalding (1998)
	Histamine H1R	I6.40E/G/A/R/K/S	Constitutive activity	Bakker (2008), Sansuk (2011)
	Rhodopsin	M6.40Y	Constitutive activity	Deupi (2012)
	AT1R	I6.40T	Increased basal activity	Parnot (2000)
	Melanocortin-4 receptor	L6.40Q	Increased basal activity	Vaisse (2000)
	TSHR	L6.40F	Increased basal activity	Tonacchera (1998)
	Lutropin-choriogonadotropic receptor	L6.40A	Constitutive activity	Fanelli (2000)
	S1P1	V6.40A/T	Reduced activation	Fujiwara (2007)
	S1P1	V6.40L	Abolished activation	Fujiwara (2007)
**6.44**	M5 muscarinic	F6.44S/T/L	Constitutive activity	Spalding (1998)
	Rhodopsin	F6.44T/V	Increased basal activity	Han (2007)
	Rhodopsin	F6.44A	No significant change in basal activity	Han (2007)
	Rhodopsin	F6.44W	Decreased basal activity	Han (2007)

	A1B adrenergic	F6.44L	Increased basal activity	Greasley(2002)
	A1B adrenergic	F6.44Y	No significant change in basal activity	Greasley (2002)
	A1B adrenergic	F6.44A/G	Decreased basal activity	Greasley (2002)
	Ghrelin receptor	F6.44Y/L	Decreased basal activity	Valenti-Hansen (2012)
	Ghrelin receptor	F6.44A	Increased basal activity	Valenti-Hansen (2012)
	GPR119	F6.44A	Decreased basal activity; loss of agonist-induced response	Valenti-Hansen (2012)
	B2AR	F6.44A	Decreased basal activity; loss of agonist-induced response	Valenti-Hansen (2012)
	NK1	F6.44A	Decreased basal activity; loss of agonist-induced response	Valenti-Hansen (2012)
	GPR39	L6.44A/Y	Decreased basal activity	Valenti-Hansen (2012)
	GPR39	L6.44F	Decreased basal activity; loss of agonist-induced response	Valenti-Hansen (2012)
	TSHR	D6.44A/E/H/Y	Constitutive activity	Porcellini (1994), Parma (1997), Russo (1996)
**7.45**	adenosine	N7.45A	Eliminated the allosteric effects of modulators but had little effect on agonist binding.	Gao (2003)
**7.52**	TSHR	L7.52V	Increased basal activity	Liu (2015)
	A2bAR	V7.52A	Reduced agonist-induced activation	Russo (1999)
	Melanocortin-1	I7.52T	Reduced basal activity	Lubrano-Berthelier (2006)
**7.53**	V2 vasopressin	Y7.53F/A	Reduced ability to bind the G-protein	Venkatakrishnan (2016)

Table C: Effects of mutations in 7.52 corresponding, for non-class A GPCRs

Generic class A residue numbering	Class	Equivalent residue numbering	Receptor	Mutants	Effect	Reference
**7.52**	B1<br>TASTE2	7.56	PTHR-1<br>TAS2R38	I7.56R<br>V296F	natural mutation generating Jansen's chrondrodysplasia; constitutive activity; change in activation of the receptor	Gensure (2001), Biarnes (2010)

MD simulation of M_2 muscarinic receptor: Setup of the system

mAchRs are expressed in the Pre-Frontal Cortex (Thiele 2013). Neuronal cells belonging to this region of the brain have a specific membrane composition (Soubias and Gawrisch 2012). Computational and experimental studies, also from our lab, showed that the composition of the membrane may influence the receptor behavior (Cao, Rossetti et al. 2015). Therefore, we built a model membrane of a neuronal cell located in the Pre-Frontal Cortex, consistent with the experimental measured composition (Table S5) (Soubias and Gawrisch 2012). All the simulations are performed with the M_2 receptor embedded in such a membrane. Notice that lipids with of concentration below 2% were not included. Their influence was considered negligible.

Lipids	Percentage
PhosphatidylCholine (PC)	16
PhosphatidylEthanolamine (PE)	16
PhosphatidylSerine (PS)	4
PhosphatidylInositol + Phosphatidic acid lipids (PI+PA)	2
Sphingomyelin	14
Cholesterol	48

Table S5: Membrane composition of a neuronal cell in the pre-frontal cortex from Ref. 19.

The CHARMM-GUI web server was employed in order to obtain a pre-equilibrated membrane (Jo, Kim et al. 2008). This is composed by 192 lipids embedded in a 110 x 110 x 30 $Å^3$ long box.

All the available structures of mAchRs lack the so-called ICL3 loop, a large cytoplasmic loop (about 130 residues). The latter might play a role in the activation mechanism of the receptor (Bernstein, Ramineni et al. 2004). ICL3 structural ensemble was predicted here by ab-initio modeling (Rodriguez, Chinea et al. 1998) because of the unavailability of reliable structural templates. Specifically, we used I-Tasser web server suite (Roy, Kucukural et al. 2010).

The AMBER99SB-ILDN force fields (Best and Hummer 2009), the Slipids (Jämbeck and Lyubartsev 2012, Jämbeck and Lyubartsev 2012), the TIP3P (Jorgensen, Chandrasekhar et al. 1983) force fields were used for the protein and ions, the lipids, and the water molecules respectively. The General Amber force field (GAFF) parameters (Wang, Wolf et al. 2004) were used for the ligands, along with the RESP atomic charge using Gaussian 09 (Frisch, Trucks et al. 2009) with the HF-6-31G* basis set (Wang, Cieplak et al. 2000, Case, Cheatham et al. 2005). MD simulations were performed using Gromacs v4.6.7 package (Van Der Spoel, Lindahl et al. 2005). MD simulation was conducted in the NPT ensemble (constant pressure and temperature) under periodic boundary conditions. A rectangular box

of 90 x 90 x 152 Å^3 along the x, y and z direction respectively has been used for M_2 in complex with antagonist and a 91 x 91 x 147 Å^3 box for M_2 in complex with agonist. The number of atoms for the latter is 128,867 and 127,733 for the M_2 in complex with the antagonist. Constant temperature and pressure conditions were achieved via independently coupling protein, lipids, solvent and ions to Nosè-Hoover thermostat (Hünenberger 2005) at 300 K and Andersen-Parrinello-Rahman Barostat (Parrinello and Rahman 1981) at 1 atm. The Particle Mesh Ewald method (Darden, York et al. 1993) was used to treat the long-range electrostatic interaction with a real space cutoff of 1.2 nm. A 1.2 nm cutoff was used for the short-range non-bonded interaction. A time step of 2 fs was set. The LINCS algorithm (Hess, Bekker et al. 1997) was applied to constrain all bonds involving hydrogen atoms. The final systems, antagonist and agonist, were then simulated for 190ns and 200ns, respectively.

References

Bakker, R. A. *et al.* Constitutively active mutants of the histamine H1 receptor suggest a conserved hydrophobic asparagine-cage that constrains the activation of class A G protein-coupled receptors. *Molecular pharmacology* **73**, 94-103, doi:10.1124/mol.107.038547 (2008).

Baranski, T. J. *et al.* C5a receptor activation. Genetic identification of critical residues in four transmembrane helices. *The Journal of biological chemistry* **274**, 15757-15765 (1999).

Biarnés X, Marchiori A, Giorgetti A, Lanzara C, Gasparini P, Carloni P, *et al.* Insights into the Binding of Phenyltiocarbamide (PTC) Agonist to Its Target Human TAS2R38 Bitter Receptor. *PLoS ONE* 5(8): e12394. doi:10.1371/journal.pone.0012394 (2010).

Bernstein, L. S., *et al.* RGS2 binds directly and selectively to the M1 muscarinic acetylcholine receptor third intracellular loop to modulate Gq/11alpha signaling. *The Journal of biological chemistry* 279(20): 21248-21256 (2004).

Best, R. B. and Hummer G. Optimized molecular dynamics force fields applied to the helix−coil transition of polypeptides. *The Journal of Physical Chemistry B* 113(26): 9004-9015 (2009).

Cao, R., *et al.* Binding of the Antagonist Caffeine to the Human Adenosine Receptor hA 2A R in Nearly Physiological Conditions. *PLoS ONE* 10(5): e0126833 (2015).

Case, D. A., *et al.* The Amber biomolecular simulation programs. *Journal of Computational Chemistry* 26(16): 1668-1688 (2005).

D'Antona, A. M. *et al.* A cannabinoid receptor 1 mutation proximal to the DRY motif results in constitutive activity and reveals intramolecular interactions involved in receptor activation. *Brain research* **1108**, 1-11 (2006).

Darden, T., *et al.* Particle mesh Ewald: An N log(N) method for Ewald sums in large systems. *The Journal of Chemical Physics* 98(12): 10089-10092 (1993)

Deupi, X. *et al.* Stabilized G protein binding site in the structure of constitutively active metarhodopsin-II. *Proceedings of the National Academy of Sciences of the United States of America* **109**, 119-124, doi:10.1073/pnas.1114089108 (2012).

Duprez, L. *et al.* Germline mutations in the thyrotropin receptor gene cause non-autoimmune autosomal dominant hyperthyroidism. *Nature genetics* **7**, 396-401, doi:10.1038/ng0794-396 (1994)

Fanelli, F. Theoretical study on mutation-induced activation of the luteinizing hormone receptor. *J Mol Biol* **296**, 1333-1351, doi:10.1006/jmbi.2000.3516 (2000).

Frisch, M. J., et al. Gaussian 09, Revision A.02. Wallingford CT (2009).

Fujiwara, Y. *et al.* Identification of the hydrophobic ligand binding pocket of the S1P1 receptor. *The Journal of biological chemistry* **282**, 2374-2385, doi:10.1074/jbc.M609648200 (2007).

Gao, Z. G., Kim, S. K., Gross, A. S., Chen, A., Blaustein, J. B., & Jacobson, K. A. Identification of essential residues involved in the allosteric modulation of the human A3 adenosine receptor. *Molecular pharmacology*, *63*(5), 1021-1031 (2003).

Gensure, R. C., Carter, P. H., Petroni, B. D., Juppner, H. & Gardella, T. J. Identification of determinants of inverse agonism in a constitutively active parathyroid hormone/parathyroid hormone-related peptide receptor by photoaffinity cross-linking and mutational analysis. *The Journal of biological chemistry* **276**, 42692-42699, doi:10.1074/jbc.M106215200 (2001).

Greasley, P. J., Fanelli, F., Rossier, O., Abuin, L. & Cotecchia, S. Mutagenesis and modelling of the alpha(1b)-adrenergic receptor highlight the role of the helix 3/helix 6 interface in receptor activation. *Molecular pharmacology* **61**, 1025-1032 (2002).

Han, M., Lou, J., Nakanishi, K., Sakmar, T. P. & Smith, S. O. Partial agonist activity of 11-cis-retinal in rhodopsin mutants. *The Journal of biological chemistry* **272**, 23081-23085 (1997).

Hess, B., et al. LINCS: A linear constraint solver for molecular simulations. *Journal of Computational Chemistry* 18(12): 1463-1472 (1997).

Ho, J. *et al.* Molecular recognition of ketamine by a subset of olfactory G protein-coupled receptors. *Science signaling* **8**, ra33, doi:10.1126/scisignal.2005912 (2015).

Hulme, E. C. GPCR activation: a mutagenic spotlight on crystal structures. *Trends in pharmacological sciences* **34**, 67-84, doi:10.1016/j.tips.2012.11.002 (2013).

Hünenberger, P. Thermostat Algorithms for Molecular Dynamics Simulations. *Advanced Computer Simulation*, Springer Berlin Heidelberg: 105-149 (2005).

Isberg, V. *et al.* GPCRDB: an information system for G protein-coupled receptors. *Nucleic acids research* **42**, D422-425, doi:10.1093/nar/gkt1255 (2014).

Jämbeck, J. P. M. and A. P. Lyubartsev Derivation and systematic validation of a refined all-atom force field for phosphatidylcholine lipids. *The Journal of Physical Chemistry B* 116(10): 3164-3179 (2012).

Jämbeck, J. P. M. and A. P. Lyubartsev An extension and further validation of an all-atomistic force field for biological membranes. *Journal of Chemical Theory and Computation* 8(8): 2938-2948 (2012).

Jo, S., *et al.* CHARMM-GUI: a web-based graphical user interface for CHARMM. *Journal of Computational Chemistry*, Wiley Subscription Services, Inc., A Wiley Company. 29: 1859-1865 (2008).

Jorgensen, W., *et al.* Comparison of simple potential functions for simulating liquid water. *J Chem Phys* 79(2): 926-935 (1983).

Kosugi, S., Hai, N., Okamoto, H., Sugawa, H. & Mori, T. A novel activating mutation in the thyrotropin receptor gene in an autonomously functioning thyroid nodule developed by a Japanese patient. *European journal of endocrinology / European Federation of Endocrine Societies* **143**, 471-477 (2000)

Liu, R., Nahon, D., le Roy, B., Lenselink, E. B. & AP, I. J. Scanning mutagenesis in a yeast system delineates the role of the NPxxY(x)(5,6)F motif and helix 8 of the adenosine A(2B) receptor in G protein coupling. *Biochem Pharmacol* **95**, 290-300, doi:10.1016/j.bcp.2015.04.005 (2015).

Lu, Z.-L. & Hulme, E. C. The functional topography of transmembrane domain 3 of the M1 muscarinic acetylcholine receptor, revealed by scanning mutagenesis. *Journal of Biological Chemistry* **274**, 7309-7315 (1999).

Lubrano-Berthelier, C. *et al.* Melanocortin 4 receptor mutations in a large cohort of severely obese adults: prevalence, functional classification, genotype-phenotype relationship, and lack of association with binge eating. *The Journal of clinical endocrinology and metabolism* **91**, 1811-1818, doi:10.1210/jc.2005-1411 (2006).

Nishihara, E. *et al.* Sporadic congenital hyperthyroidism due to a germline mutation in the thyrotropin receptor gene (Leu 512 Gln) in a Japanese patient. *Endocrine journal* **53**, 735-740 (2006).

Parma, J. *et al.* Diversity and prevalence of somatic mutations in the thyrotropin receptor and Gs alpha genes as a cause of toxic thyroid adenomas. *The Journal of clinical endocrinology and metabolism* **82**, 2695-2701, doi:10.1210/jcem.82.8.4144 (1997).

Parnot, C. *et al.* Systematic identification of mutations that constitutively activate the angiotensin II type 1A receptor by screening a randomly mutated cDNA library with an original pharmacological bioassay. *Proceedings of the National Academy of Sciences of the United States of America* **97**, 7615-7620, doi:10.1073/pnas.110142297 (2000).

Parrinello, M. and A. Rahman Polymorphic transitions in single crystals: A new molecular dynamics method. *Journal of Applied Physics* 52(12): 7182-7190 (1981).

Porcellini, A. *et al.* Novel mutations of thyrotropin receptor gene in thyroid hyperfunctioning adenomas. Rapid identification by fine needle aspiration biopsy. *The Journal of clinical endocrinology and metabolism* **79**, 657-661, doi:10.1210/jcem.79.2.8045989 (1994).

Rodriguez, R., *et al.* Homology modeling, model and software evaluation: three related resources. *Bioinformatics* 14(6): 523-528 (1998).

Roy, A., *et al.* I-TASSER: a unified platform for automated protein structure and function prediction. 5(4): 725-738 (2010).

Russo, D. *et al.* Thyrotropin receptor gene alterations in thyroid hyperfunctioning adenomas. *The Journal of clinical endocrinology and metabolism* **81**, 1548-1551, doi:10.1210/jcem.81.4.8636365 (1996).

Russo, D. *et al.* A Val 677 activating mutation of the thyrotropin receptor in a Hurthle cell thyroid carcinoma associated with thyrotoxicosis. *Thyroid : official journal of the American Thyroid Association* **9**, 13-17 (1999)

Sansuk, K. *et al.* A structural insight into the reorientation of transmembrane domains 3 and 5 during family A G protein-coupled receptor activation. *Molecular pharmacology* **79**, 262-269, doi:10.1124/mol.110.066068 (2011).

Soubias, O. and K. Gawrisch The role of the lipid matrix for structure and function of the GPCR rhodopsin. *BBA - Biomembranes* 1818(2): 234-240 (2012).

Spalding, T. A., Burstein, E. S., Henderson, S. C., Ducote, K. R. & Brann, M. R. Identification of a ligand-dependent switch within a muscarinic receptor. *Journal of Biological Chemistry* **273**, 21563-21568 (1998).

Taddese, Bruck, et al. "Do plants contain g protein-coupled receptors?. *Plant physiology* **164.1** (2014)

Tao, Y.-X., Abell, A. N., Liu, X., Nakamura, K. & Segaloff, D. L. Constitutive activation of G protein-coupled receptors as a result of selective substitution of a conserved leucine residue in transmembrane helix III. *Molecular Endocrinology* **14**, 1272-1282 (2000).

Thiele, A. Muscarinic Signaling in the Brain. *Annual Review of Neuroscience* 36(1): 271-294 (2013).

Tonacchera, M. *et al.* Hyperfunctioning thyroid nodules in toxic multinodular goiter share activating thyrotropin receptor mutations with solitary toxic adenoma. *The Journal of clinical endocrinology and metabolism* **83**, 492-498, doi:10.1210/jcem.83.2.4559 (1998).

Trulzsch, B. *et al.* Detection of thyroid-stimulating hormone receptor and Gsalpha mutations: in 75 toxic thyroid nodules by denaturing gradient gel electrophoresis. *J Mol Med (Berl)* **78**, 684-691 (2001).

Vaisse, C. *et al.* Melanocortin-4 receptor mutations are a frequent and heterogeneous cause of morbid obesity. *The Journal of clinical investigation* **106**, 253-262, doi:10.1172/JCI9238 (2000).

Valentin-Hansen, L., Holst, B., Frimurer, T. M. & Schwartz, T. W. PheVI: 09 (Phe6. 44) as a sliding microswitch in seven-transmembrane (7TM) G protein-coupled receptor activation. *Journal of Biological Chemistry* **287**, 43516-43526 (2012).

Van Der Spoel, D., *et al.* GROMACS: Fast, flexible, and free. *Journal of Computational Chemistry* 26(16): 1701-1718 (2005).

Venkatakrishnan, A. J., *et al.* Diverse activation pathways in class A GPCRs converge near the G-protein-coupling region. *Nature* **536**.7617, 484-487 (2016).

Wang, J. M., *et al.* How well does a restrained electrostatic potential (RESP) model perform in calculating conformational energies of organic and biological molecules? *Journal of Computational Chemistry* 21(12): 1049-1074 (2000).

Wang, J. M., *et al.* Development and testing of a general amber force field. *Journal of Computational Chemistry* 25(9): 1157-1174 (2004).
